# Editorial

**Published:** 1853

**Authors:** 


### EDITORIAL.

LOUISVILLE, May 12th, 1854.

After so long a time, we are able at length to present the *Western Journal of Medicine and Surgery*, for November and December, to our readers. This double number completes the volume for 1853, and fills up another series of the Journal. The *new series* was commenced in January, and the first number has been forwarded to all our subscribers.

We regret the delay which has attended the publication of this concluding number more than our readers can, but it has depended upon circumstances beyond our control. All the materials of the former printer and proprietor of the Journal were consumed by fire, in November, and it was necessary to procure a new publisher. Much time was wasted in making preparations to begin it, and much unavoidable delay has occurred since it was commenced, so that, instead of having it out, as we hoped to do, in the winter, summer will be upon us by the time it appears. All that we can now do, is to cast ourselves upon the indulgence of our readers, and promise that, if our efforts can avail, things shall be managed better in future.

We have given proof, we think, that this promise is not an idle one. Five numbers of our new volume are already before our readers, and their testimony, so far as it has come to us, is, that we have been "improved by fire;" we are sure that our new issue must strike every one as an improvement upon our former series.

It was somewhat doubtful, at the close of last year, whether the *Western Journal* would be continued. We felt inclined to retire from our arduous post. We flattered ourselves that we had labored for the profession long enough in this vocation. Other duties were pressing, and other employments were more profitable; but it was deemed by our friends that the Journal ought not to be suspended, and we were pressed to continue in its service. We certainly find it more pleasant to go on since we have secured the active co-operation of a sufficient body of able contributors, and been able to get the work executed in a style more worthy of its patronage and literary support.

In concluding another series of our work, we may be permitted to say a word about the course of the *Journal*. We have endeavored to make it impartial. Truth has been our object at all times, in every discussion, upon every subject. It has been our wish to be temperate, and while setting forth our views freely and plainly, to avoid over-stepping the line of decorum. We know how difficult it is in excited controversy to use language always becoming, but in looking back over all that we have written, we have not found many words to regret. In all our controversies, some of which without any fault of ours have been too personal, we have kept constantly in mind, first, what was due to ourselves, and, then, what we owed to our profession. We have criticized severely, where we thought error demanded sharp rebuke, but we have always been careful to do so in language in use among gentlemen.

We have a noble, an exalted profession, but its dignity and its interests are not to be sustained and advanced without watchfulness and labor on the part of its members.

All, in this world, is not progress; the tendency is not always in the direction of improvement. Medicine and the medical profession are assailed by dangers which must be met and averted. There is not merely quackery in its myriad shapes which we have to oppose, but there is a supineness on the part of physicians which must be aroused if we would see the profession advance to a point of renown which it ought to attain. Medical men, in our country, we presume in all countries, are generally far from taking as much interest as they ought in the advancement of our common calling. They neglect to read; they neglect the periodical literature of their profession; they neglect its societies and associations; in a word, they do not work, save in the field of practice. They work hard there. They are faithful, laborious practitioners, but they do not continue students, and they lend no helping hand to the enterprises designed to elevate and push forward the profession. There is but little co-operation—but little concert of action. Now, all this it is the business of the medical press to reform. We must have a more reading profession, more concert, more *esprit du corps*, and the medical journals must awaken it. From all quarters, we hear complaints of small attendance on medical societies, failure of committees to report, failure of everything that imparts life and usefulness to such societies; all of which indicates that the medical journals have an important function to perform. There is a tendency in all times to get "out of joint," and it is the duty of good and wise men to exert themselves to set them right.

In this view, we hope it will be conceded that the motive which impels us to continue our journal is a laudable one, and we trust that we are addressing many who will enter into our feelings and give us their cordial support. There is nothing selfish in this appeal. We have no pecuniary interest in our journal, but are devoting ourselves to the enterprise, as we have pursued it from the first, at a sacrifice. We are actuated by but one motive in continuing our connection with it—a desire to promote the interests of the profession.

